# Effects of n-3 PUFA supplementation on oocyte in vitro maturation in mice with polycystic ovary syndrome

**DOI:** 10.1186/s13048-023-01162-w

**Published:** 2023-04-29

**Authors:** Rujun Ma, Shuxian Wang, Mengqi Xue, Hong Zhang, Zhaowanyue He, Kadiliya Jueraitetibaike, Xie Ge, Li Chen, Bing Yao

**Affiliations:** 1 State Key Laboratory of Reproductive Medicine, Nanjing Medical University, Nanjing, 211166 China; 2 Center of Reproductive Medicine, Affiliated Jinling Hospital, Medical School, Nanjing University, Nanjing, 210002 China; 3 Changzhou Maternal and Child Health Care Hospital, Changzhou Medical Center, Nanjing Medical University, Changzhou, 213000 China

**Keywords:** n-3 PUFA, Polycystic ovary syndrome, Oocyte in vitro maturation, Oxidative stress

## Abstract

n-3 PUFAs are classic antioxidant that can be used to treat follicular dysplasia and hyperinsulinemia caused by excessive oxidative stress in PCOS women. To investigate the effect of n-3 PUFA supplementation on the oocyte quality of polycystic ovary syndrome (PCOS) mice during in vitro maturation, a PCOS mouse model was established by dehydroepiandrosterone (DHEA). The GV oocytes of the control and PCOS groups were collected and cultured in vitro with or without n-3 PUFAs. After 14 h, the oocytes were collected. Our data demonstrated that the oocyte maturation rate of PCOS mice significantly increased after the addition of 50 µM n-3 PUFAs. The results of immunofluorescence showed that the abnormal rates of spindles and chromosomes in the PCOS + n-3 PUFA group were lower than those in the PCOS group. The mRNA expression of an antioxidant-related gene (*Sirt1*) and DNA damage repair genes (*Brca1/Msh2*) was found to be significantly rescued after n-3 treatment. Additionally, the results of living cell staining showed that the addition of n-3 PUFAs could reduce the levels of reactive oxygen species and mitochondrial superoxide in PCOS oocytes. In conclusion, the addition of 50 µM n-3 PUFAs during the in vitro maturation of PCOS mouse oocytes can improve the maturation rate by reducing the level of oxidative stress and the rate of spindle/chromosome abnormalities, providing valuable support during the IVM process.

**Supplementary Information:**

The online version contains supplementary material available at 10.1186/s13048-023-01162-w.

## Introduction

Oocyte quality can affect embryo development, embryo survival and pregnancy success rate [1]. The developmental potential of oocytes to form embryos comes from their maturation process [2]. In some cases, immature oocytes can also be cultured and matured in vitro and used to form embryos through assisted reproductive technology. Inspired by animal research, Trounson et al. applied in vitro maturation (IVM) technology to PCOS patients for the first time and proposed that it could be used as an alternative to assisted reproduction that is more beneficial to patients [3]. A clinical study found that the success rate of IVM in women with a high sinus follicle count, especially those with PCOS, was higher than that in women with normal functional ovarian reserve [4].

PCOS is a common reproductive endocrine disease affecting approximately 6%~10% of women of childbearing age. Infertility patients with PCOS are mainly characterized by follicular dysgenesis, hyperinsulinemia and insulin resistance, which adversely affect oocyte maturation, development and fertilization [5]. By comparing the transcriptomes of oocytes from healthy people and PCOS patients, mitochondrial dysfunction was found to be one of the main differences in oocyte development [6]. Increased ROS, decreased ATP production and apoptosis are the basic features of mitochondrial dysfunction. Research has shown that compared with healthy women, women with PCOS are in a pathological state of oxidative stress, and the ROS level in the ovary is increased [7]. An imbalance between free radicals and antioxidants in the follicular fluid of PCOS patients can lead to oxidative stress, abnormal follicular formation, and oocytes inherent defects [8]. Therefore, it is very important to find drugs to reduce oxidative stress levels, improve mitochondrial function, and improve oocyte maturation rate and quality in PCOS patients for successful clinical pregnancy.

Antioxidants can reduce ROS levels and oocyte apoptosis mediated by oxidative stress, thereby protecting female fertility [9]. As a classic antioxidant, n-3 PUFAs can be used to treat follicular dysplasia and hyperinsulinemia caused by excessive oxidative stress in PCOS women [10]. Studies have shown that n-3 PUFAs promote the biosynthesis of estradiol and progesterone in granulosa cells of PCOS by activating the PI3K/AKT pathway [11]. A prospective clinical study showed that serum levels of n-3 PUFAs were positively correlated with the clinical pregnancy rate and live birth rate in patients receiving assisted reproductive treatment [12]. Numerous animal and clinical experiments have shown that a diet rich in n-3 PUFAs can improve female fertility [13] by improving oocyte quality [14, 15], affecting embryo implantation [16] and regulating reproductive hormone levels [17].

To better benefit patients from IVM, animal experiments can be used to study ways to improve the success rate of IVM. Therefore, in this study, the influence of n-3 PUFAs in the treatment of PCOS was determined by oocyte culture in vitro using a mouse model, and the mechanism of action was explored by detecting oxidative stress levels, providing a certain experimental basis for the clinical improvement of oocyte quality in PCOS patients.

## Results

### The validation of PCOS model construction

**Fig. 1:**
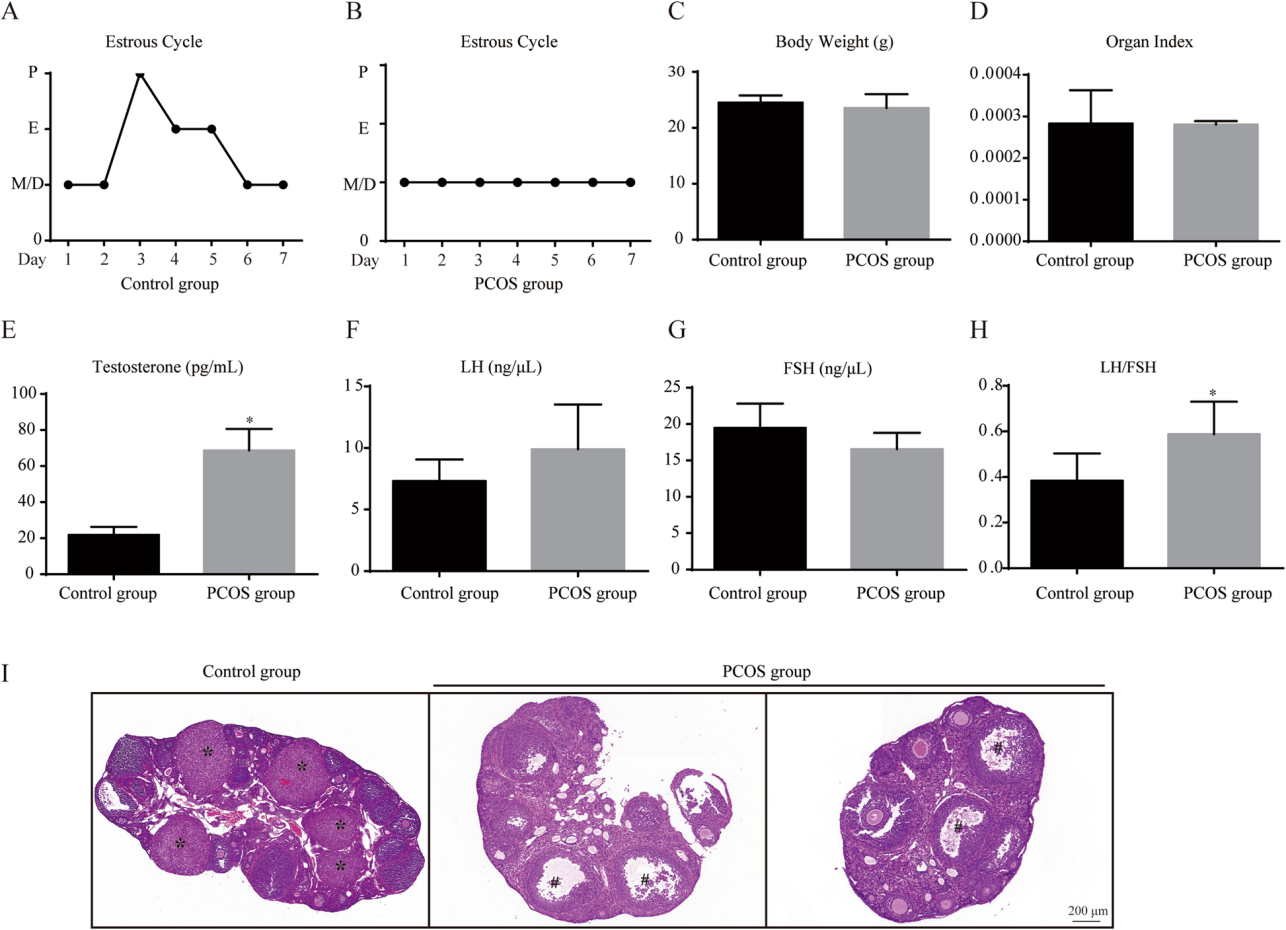
Estrous cycle changes and alterations of serum hormones in two representative mice from the control and PCOS groups. **A**, **B** P: proestrus; E: estrus; M: metestrus; D: dioestrus; **C** The body weight of mice in both groups was calculated; **D** Organ mass ratio of ovary to body weight; **E**-**G** Statistics of serum testosterone, LH and FSH content. **H** Ratio of LH to FSH. **I** Representative images of ovarian sections. Scale bars: 200 μm. The asterisk (*) represents the corpus luteum, and the pound sign (#) represents the ovary vacuoles. Data are expressed as the mean ± SD, *p < 0.05 vs. control

Vaginal smear evaluation showed a natural cycle in control mice consisting of estrus, proestrus, metestrus, and diestrus stages. However, constant pseudo-diestrous was observed in the PCOS model, which indicated the lack of cyclic oestrus (Fig. 1A-B). Compared with the control group, PCOS mice showed a significant increase in testosterone (*p* < 0.0001), LH/FSH ratios (*p* = 0.0399), as detailed in Fig. 1. H&E staining was used to determine the alteration of follicle morphology in different groups (Fig. 1I). Compared with the control group, PCOS group had more cystic follicles and less corpus luteum. The above results suggested that PCOS modeling was basically successful.

Furthermore, we measured several oxidative stress markers, such as superoxide dismutase (SOD) activity, protein carbonyl and malondialdehyde (MDA) in control and PCOS ovaries. Reduced SOD enzyme activity, increased MDA and protein carbonyl levels suggested that PCOS mice suffered high levels of oxidative stress (Supplementary Fig. 1).

### Effect of n-3 PUFAs on PCOS oocyte maturation in vitro 

**Fig. 2:**
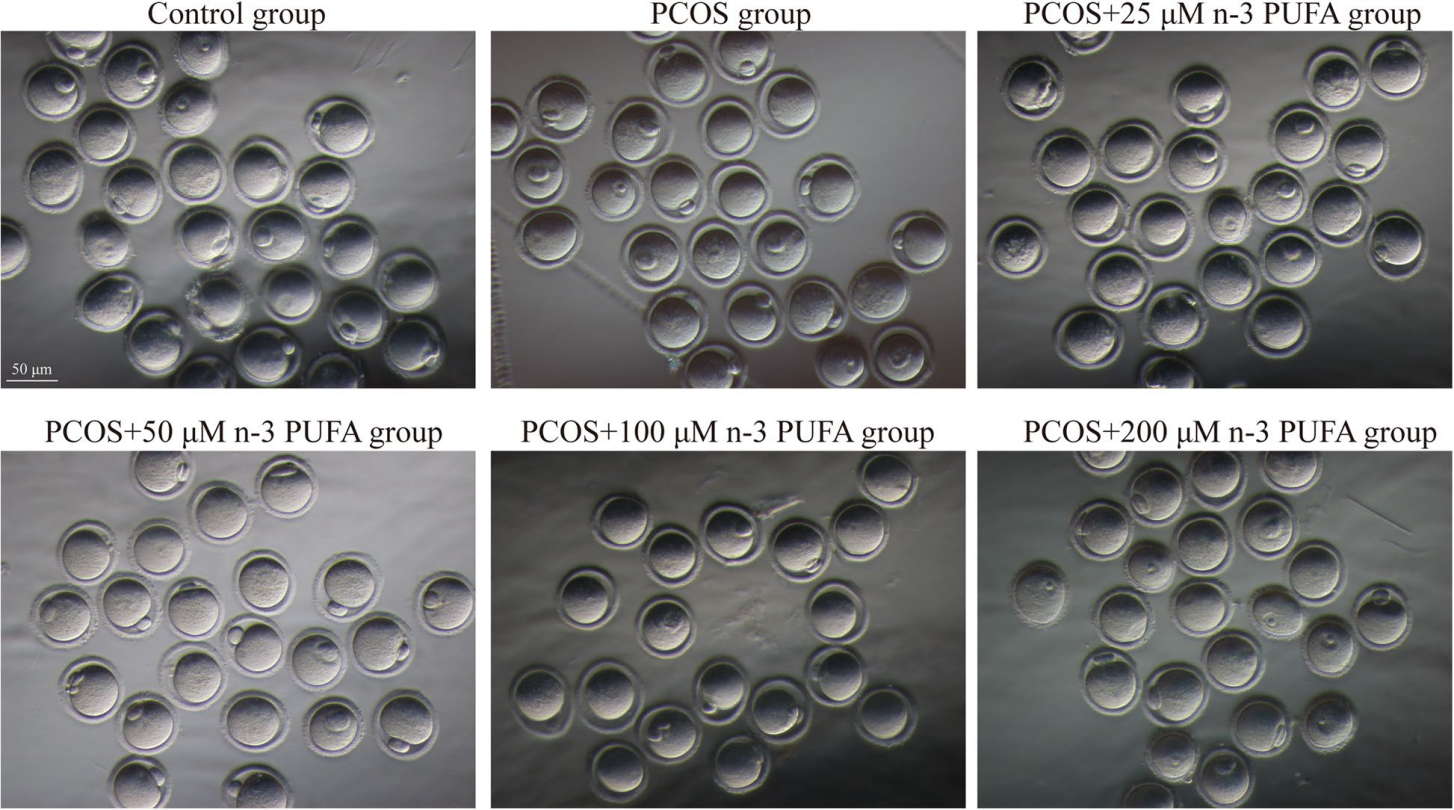
Representative phase contrast images of MII oocytes derived from diverse groups. Images were acquired with a camera on a stereomicroscope (IX73, Olympus Corporation, Japan) with the x20 objectives

During IVM culture, the first polar body expulsion rate (MII rate) of PCOS mice was significantly decreased compared with that of the normal group (*p* = 0.0103). To confirm the appropriate concentration of n-3 PUFAs added to the IVM culture medium, we added 25 µM, 50 µM, 100 µM, and 200 µM n-3 PUFAs to culture GV stage oocytes from PCOS mice and counted their maturation rates after 14 h. The results showed that 50 µM n-3 PUFAs could rescue the decrease in oocyte maturation rate caused by PCOS (*p* = 0.0271) (Fig. 2). Therefore, 50 µM n-3 PUFAs were selected for subsequent experiments, as detailed in Table 1. The first polar body expulsion rate (MII rate) of oocytes in the PCOS + 50 µM n-3 PUFA group was significantly higher than that in the PCOS group, suggesting that the addition of n-3 PUFAs to IVM culture medium could significantly increase the oocyte maturation rate in PCOS mice (Table 1).

**Table 1:** The Pb1 extrusion rate after n-3 PUFA treatment

Groups	Pb1 extrusion rate(%)
**Control group**	79.39 ± 1.925
**PCOS group**	66.96 ± 1.932^a^
**PCOS + 25 µM n-3 PUFA group**	68.30 ± 4.219^a^
**PCOS + 50 µM n-3 PUFA group**	78.39 ± 2.743^b^
**PCOS + 100 µM n-3 PUFA group**	58.58 ± 1.899^ab^
**PCOS + 200 µM n-3 PUFA group**	38.63 ± 3.679^ab^

Compared with the control group

^a^*p* <0.05; compared with the PCOS group

^b^*p*<0.05

### Effect of n-3 PUFAs on spindle/chromosome structure in PCOS oocytes 

**Fig. 3:**
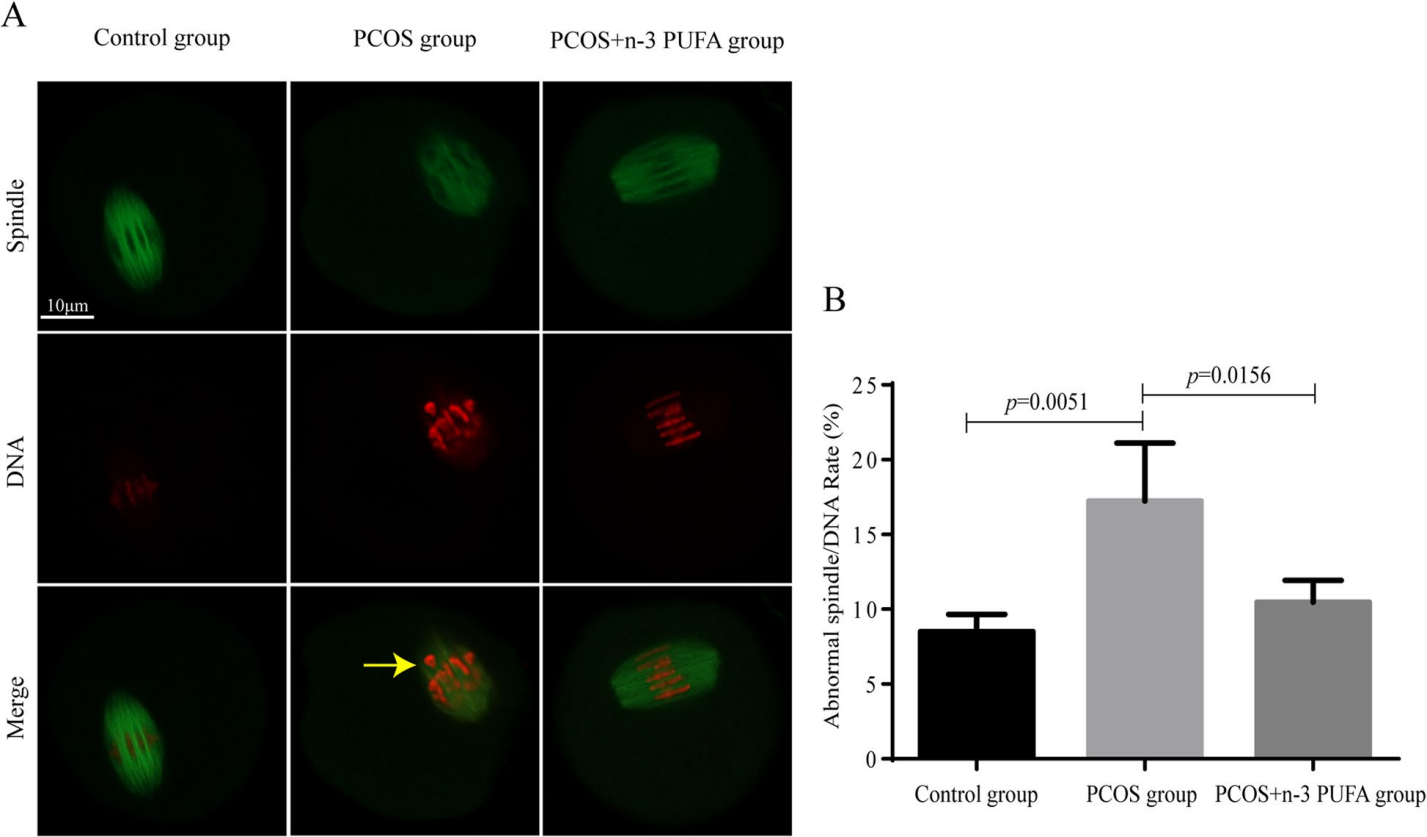
Effects of n-3 PUFAs on the abnormal rate of spindle/chromosome structure in PCOS. **A** Normal and abnormal spindle/chromosome structure shown by immunofluorescence staining; **B** proportion of oocytes with abnormal spindle/chromosome in the three groups

The above data suggested the possibility that n-3 PUFA treatment may affect the meiotic apparatus in oocytes. To investigate the regulatory mechanism of n-3 PUFAs during meiosis, control, PCOS and n-3 PUFA-treated PCOS oocytes were immunolabeled with anti-tubulin antibody to visualize the spindle and were costained with PI to bind chromosomes. As shown in Fig. 3, during IVM culture, the proportion of abnormal spindle distribution of PCOS mice was significantly increased compared with that in the normal group. After the addition of 50 µM n-3 PUFAs, the proportion of abnormal spindle distribution was significantly reduced.

### The effect of n-3 PUFAs on the expression of oxidative stress related genes in oocytes of PCOS mice

**Fig. 4:**
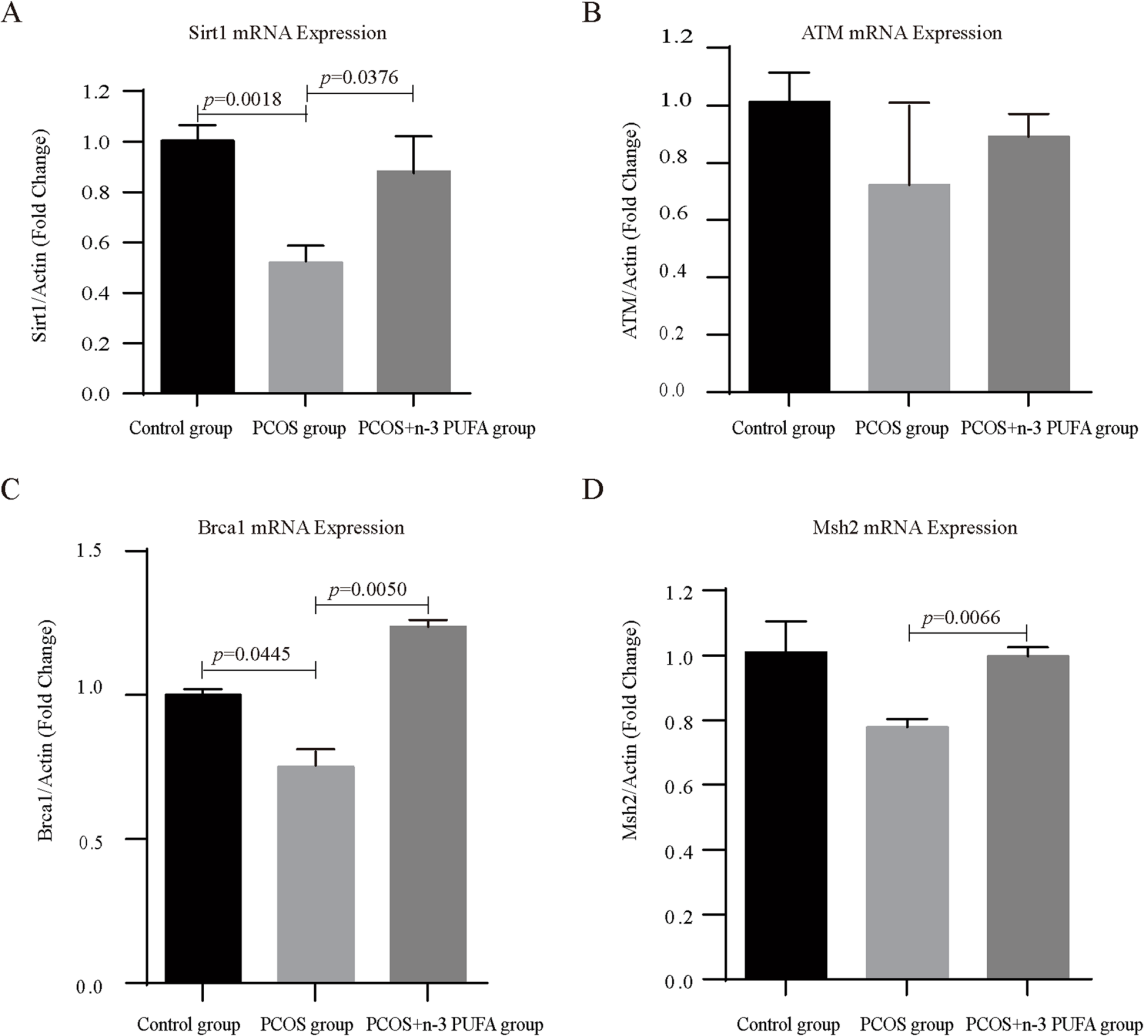
The relative mRNA expression of *Sirt1*, *ATM*, *Brca1* and *Msh2* in control, PCOS and PCOS + n-3 PUFA group oocytes. The relative mRNA levels were determined by RT-qPCR. Data are expressed as the mean ± SD

Sirt1 has been reported to be a key regulator of the antioxidant defense system and is associated with oocyte quality. Therefore, we checked whether *Sirt1* expression in oocytes was accordingly changed in response to PCOS and n-3 PUFA treatment. The mRNA expression levels of *Sirt1* in oocytes of the control group, PCOS group and PCOS + n-3 PUFA group were detected. For RNA extraction, GV oocytes in different groups were cultured for 14 h. The results showed that the expression levels of *Sirt1* mRNA in oocytes of the PCOS group were significantly lower than those of the control group, and n-3 PUFA treatment rescued the *Sirt1* mRNA expression level in PCOS oocytes (Fig. 4A). The mRNA levels of other key antioxidant enzymes, such as *SOD3*, *CAT* and *Nrf2*, and lipid peroxidation marker *GPX4* has also been detected. All results showed no significantly difference, except for the changes of *SOD3* mRNA level between PCOS and PCOS + n-3 PUFA group (Supplementary Fig. 2). These results suggested that Sirt1 may be the key target for n-3 PUFA to rescue PCOS oocytes.

### The effect of n-3 PUFAs on DNA damage repair in oocytes of PCOS mice

To investigate the deep-seated regulatory mechanism of n-3 PUFAs in PCOS oocytes, the mRNA expression levels of DNA damage repair genes (*Brca1*, *ATM* and *Msh2*) in oocytes of the control group, PCOS group and PCOS + n-3 PUFA group were detected. The results showed that the mRNA expression levels of *Brca1* in oocytes of the PCOS group were significantly lower than those of the control group. Although the mRNA expressions of *ATM* and *Msh2* in PCOS oocytes did not decrease significantly, they also showed a downward trend. Treatment with n-3 PUFAs rescued the mRNA expression levels of *Brca1* and *Msh2* in PCOS oocytes. Detailed results are shown in Fig. 4B-D.

### Effect of n-3 PUFAs on oxidative stress in oocytes of PCOS mice

**Fig. 5:**
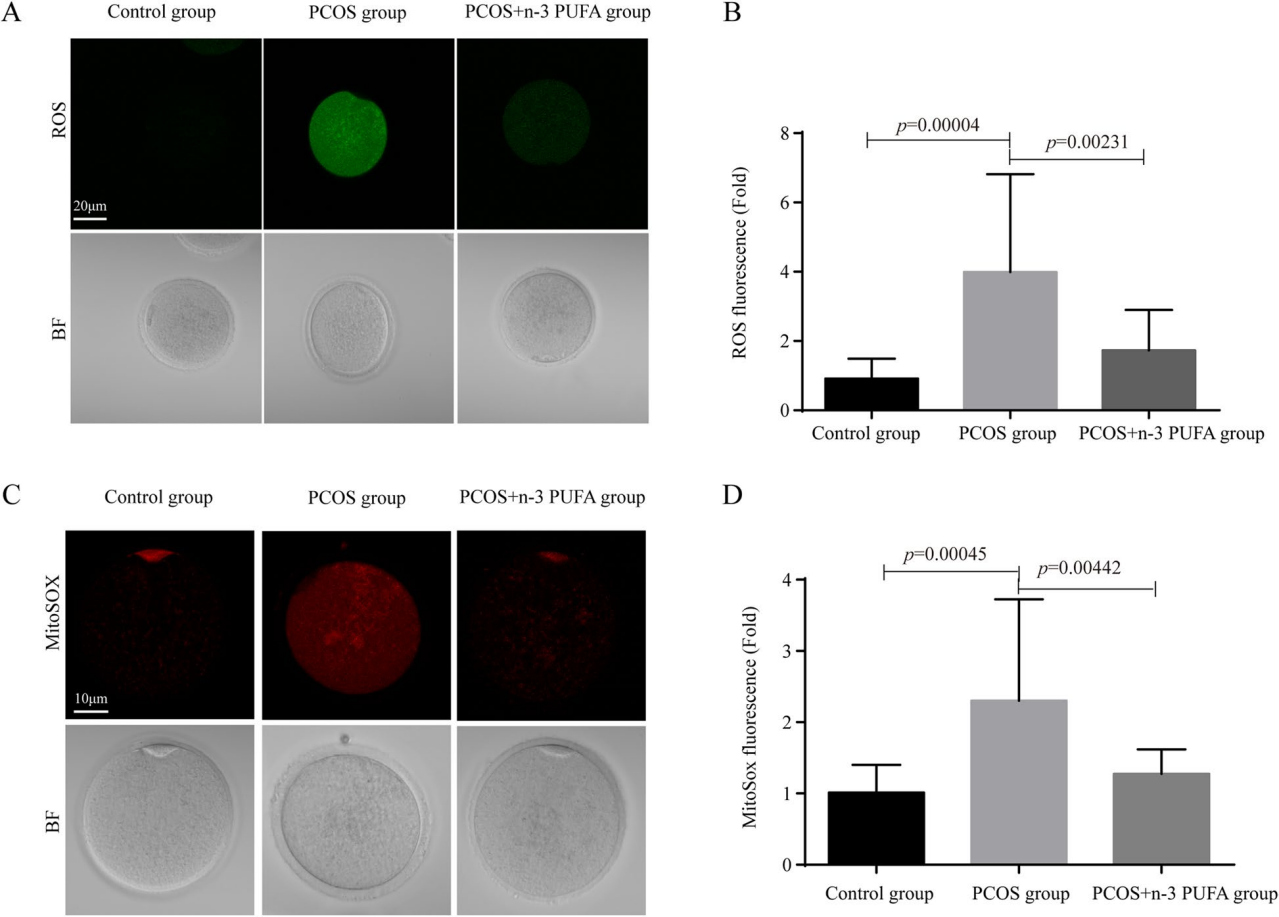
Effects of n-3 PUFAs on oxidative stress in PCOS mouse oocytes. **A** Changes in reactive oxygen species levels among the three groups; **B** Quantification statistics of reactive oxygen species levels among the three groups (*n* = 25 for each group); **C** Changes in mitochondrial superoxide levels among the three groups; **D** Quantification of mitochondrial superoxide levels among the three groups (*n* = 25 for each group). Data are expressed as the mean ± SD. Statistical analyses were carried out using one-way ANOVA followed by Games-Howell method

The effect of n-3 PUFAs on the neutralization of free radicals and mitochondrial oxidation was studied. During IVM culture, ROS production in PCOS mice was significantly enhanced compared with the control group. ROS in oocytes of the PCOS + n-3 PUFA group was significantly attenuated compared with PCOS. MitoSOX also showed the same trend. Taken together, the addition of n-3 PUFAs to the IVM culture medium could significantly reduce oxidative stress levels in the oocytes of PCOS mice. Detailed results are shown in Fig. 5.

## Discussion

Compared with traditional in vitro fertilization techniques, IVM could avoid the use of gonadotropins for ovarian stimulation to directly obtain mature MII eggs, reduce costs, and avoid the occurrence of ovarian hyperstimulation syndrome. As a result, IVM is widely used in clinical practice as a new assisted reproductive technology. Because PCOS patients are at high risk of developing ovarian hyperstimulation syndrome, IVM is more applicable for these patients [4]. Poor oocyte quality and low success rate of IVM are important challenges for successful pregnancy by in vitro fertilization.

The balance between ROS and antioxidant levels within the ovary is important for maintaining female fertility [18]. The oxidative stress-induced mitochondrial pathway plays a major role in apoptosis in oocytes. During IVM, the first polar body extrusion rate would be compromised if ROS levels were too high. Excessive accumulation of ROS damages oocytes, resulting in mitochondrial dysfunction, spindle abnormalities, apoptosis, and impaired development [19]. It has been reported that excessive accumulation of ROS in PCOS mouse models leads to increased abnormal oocyte morphology, reduced oocyte maturation rate, and reduced oocyte quality [20]. Our findings also yielded similar results, with PCOS mice having significantly higher oocyte ROS levels than controls (*p* < 0.05). Animal studies have shown that supplementation with the antioxidant melatonin during IVM was beneficial in rescuing the deleterious effects of ROS on oocytes in PCOS mice [21]. This study and our experimental results indicated that supplementation with antioxidants in IVM culture medium was able to improve oocyte quality in PCOS mice.

Omega-3 polyunsaturated fatty acids (n-3 PUFAs), which are composed of eicosapentaenoic acid (EPA) and docosahexaenoic acid (DHA), have a higher calorifific value compared to other dietary supplements, and their use should be carefully evaluated in obese or overweight patients to avoid negative impact on metabolic alterations, which are common in PCOS women [22]. Adding n-3 PUFAs in IVM can avoid this risk. Although the beneficial effects of DHA and EPA have been realized, several studies have demonstrated that PUFA concentrations impair oocyte nuclear maturation. High dose of DHA and EPA have been reported play harmful effects on oocyte developmental competence in vitro [23, 24]. An excessive dose of n-3 PUFAs can affect lipid metabolism gene expression [23] and lead to increased sensitivity to ferroptosis [25]. Thus, the appropriate dosage of n-3 PUFAs needs to be determined. According to the previous research in our lab, 10 to 200 µM n-3 PUFAs have been used [26, 27]. In this study, we constructed a mouse PCOS model and found that the addition of 50 µM n-3 PUFAs could significantly increase the maturation rate of the oocytes from PCOS mice via IVM culture (*P* < 0.05), suggesting that the supplementation of n-3 PUFA in vitro culture medium could also improve oocyte quality in PCOS mice.

Wan et al. found that Sirt1 could be an important biomarker in PCOS treatment through single-cell sequencing data analysis of oocytes from PCOS patients [28]. Downregulation of the Sirt1/Nrf2 pathway, a classical antioxidative stress pathway, has been found to play an important role in oocyte quality decline and spindle morphology abnormalities caused by aging [29]. Inhibition of Sirt1 expression could cause oocyte maturation disorders and abnormal spindle assembly [30]. The downregulation of Sirt1 expression detected in our study may be responsible for the increased rate of abnormal spindle assembly and increased ROS in oocytes of PCOS mice. In a variety of disease models, the addition of n-3 PUFAs was able to exert their rescue by upregulating the expression of Sirt1 [31, 32], and DHA and EPA could also exert their function by upregulating Sirt1 [33]. Our study confirmed that the addition of n-3 PUFAs could also restore Sirt1 expression in oocytes from PCOS model mice during IVM. Loss of Sirt1 was able to accelerate DNA damage [34] and increase DNA damage and DNA susceptibility to oxidative stress in PCOS women [35]. Therefore, we focused on DNA damage repair-related genes.

BRCA1, ATM, and msh2 are DNA damage repair-related genes associated with oxidative stress [36–39], but alterations of these genes have not been reported in PCOS. Numerous studies have indicated that BRCA1 also regulates oxidative stress itself: loss of BRCA1 increases cellular ROS, while overexpression of BRCA1 inhibits ROS production [40]. The addition of n-3 PUFAs has been reported to increase Brca1 expression in rat mammary tumors [41]. Msh2 is involved in repairing ROS-induced base abnormalities [42]. ATM, preferentially activated by DNA double-strand breaks, has been shown to be a sensor of oxidative stress, with ATM-deficient cells being more susceptible to oxidative stress inducers and DNA-damaging agents. ATM-deficient cells accumulate ROS, suggesting that ATM is essential in cellular oxidative stress defense programs [39]. ATM depletion results in sustained DNA damage, PARP activation, and NAD + depletion. NAD + deletion resulted in Sirt1 inactivation, mitochondrial phagocytic defects, and mitochondrial dysfunction [43]. Therefore, we hypothesized that in PCOS oocytes, the decrease in DNA damage repair genes could be one of the reasons for the increase in ROS, and n-3 PUFAs might exert their function by regulating DNA damage repair genes. However, the reasons for the reduced expression of DNA damage repair genes in oocytes induced by PCOS and how n-3 PUFAs regulate DNA damage repair genes remain to be further investigated.

**Fig. 6:**
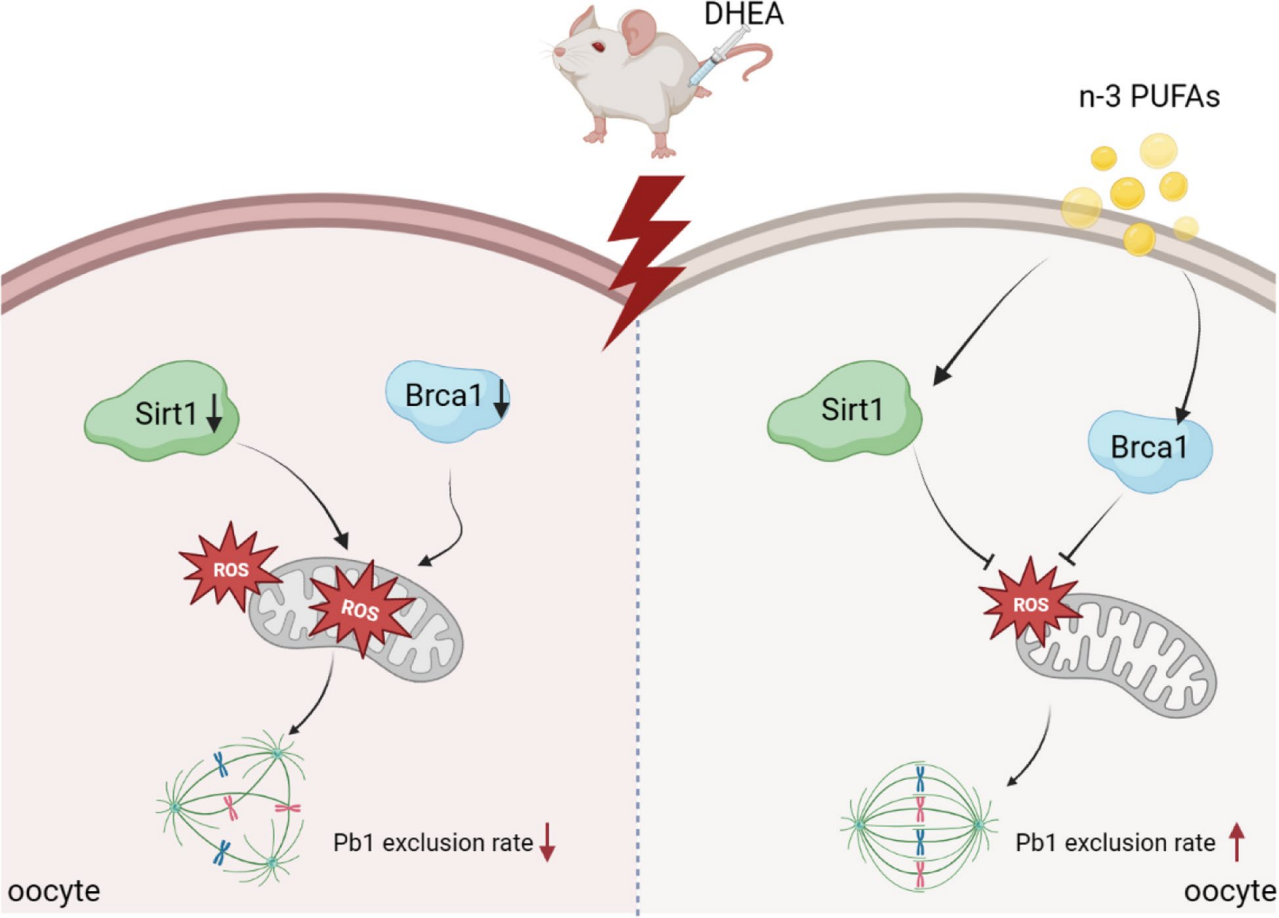
Diagram illustrating the proposed pathway mediating the beneficial effects of n-3 PUFAs on oocyte phenotypes induced by PCOS. n-3 PUFAs could improve PCOS oocyte quality during IVM by regulating the antioxidant gene *Sirt1* and DNA damage repair-related gene *Brca1* to reduce ROS levels and MitoSOX levels

In conclusion, our study showed that n-3 PUFAs, as a classical antioxidant, could improve oocyte quality in PCOS mice by regulating the antioxidant gene *Sirt1* and DNA damage repair-related genes to reduce ROS levels and MitoSOX levels (Fig. 6). Based on the above results, this study preliminarily investigated the effect of n-3 PUFAs on oocyte quality improvement in PCOS mice, laying the foundation for the clinical application of n-3 PUFAs in IVM of PCOS patients.

## Materials and methods

### Animals and diet

All experiments were reviewed by the Ethics Committee of the Jinling Hospital (2021DZGKJDWLS-00119) and were performed according to institutional guidelines. Three-week-old female ICR mice were fed clean-grade food at room temperature (18 ~ 24 °C) and exposed to light at 7:00 ~ 19:00 every day. After 3 days, the mice were randomly divided into the control group and PCOS group. DHEA (Dehydroepiandrosterone, cat. no., 2017020, Nanjing Kangmanlin Biomedical Technology, China) was dissolved in corn oil (cat. no., 20200502, Shandong Ruisheng Pharmaceutical Excipients, China). The PCOS model was induced by subcutaneous injection at 9:00 am every day, while the control group was injected with the same dose of corn oil subcutaneously at 9:00 am every day. Daily vaginal smears were taken on the 21st day to observe the estrous cycle of mice in each group for 7 days to determine whether the PCOS model was successfully constructed.

### Determination of serum hormones

After the intervention, all mice fasted at 20:00 am at the end of the intervention day and were anesthetized with 10% chloral hydrate (Sigma-Aldrich, USA) by intraperitoneal injection at 8:00 am the next day. Serum was extracted, and LH, FSH and testosterone concentrations were determined by ELISA. All procedures were performed in strict accordance with the kit instructions.

### Oocyte collection and culture

Collected mouse GV-stage oocytes were placed in M2 medium (cat. no., M7161, Sigma-Aldrich, USA,) for in vitro culture, and the first polar body extrusion rate was calculated after 14 h of culture. Pufas of 0, 25, 50, 100, and 200 µM n-3 PUFAs (cat. no., 210100, OMEGA 3 TREASURE, China) were added to the M2 culture medium of PCOS mouse oocytes, and the first polar body extrusion rate was calculated after 14 h of culture.

### Determination of ROS and MitoSOX

The mitochondrial superoxide indicator MitoSOX Red (cat. no., M36008, Thermo Fisher Technology, USA) and reactive oxygen species (ROS) assay kit (chemical fluorescence method) (no. E004-1-1, Nanjing Jiancheng Bioengineering Research Institute, China) were used to determine the production of reactive oxygen species (ROS) and superoxide in the mitochondria of oocytes. Oocytes were moved into DPBS with 0.1% BSA diluted in ROS (1:1000) or MitoSOX (1:500) for 30 min in a 37 °C incubator, and was washed by DPBS with 0.1% BSA twice, 3–5 min each time, and then moved into a glass pan after washing. Zeiss laser scanning confocal microscopy (LSM 810, Zeiss, Germany) was used to observe and record the fluorescence images.

### Immunofluorescence

Oocytes were fixed in 4% PFA for 30 min and permeabilized with 0.5% Triton X-100 for 20 min. Oocytes were then blocked in 1% BSA for 1 h and incubated overnight using antibody dilutions (α-tubulin-FITC, 1:250) at 4 °C. After washing, PI (1:350) was used for 10 min at room temperature and examined under a laser scanning confocal microscope (LSM 810, Zeiss, Germany) equipped with a 40x objective. Fluorescence intensity was quantified using ImageJ software (NIH).

### Quantitative real-time PCR

After 14 h in vitro culture, total RNA of different groups were isolated from 30 oocytes using an RNA Isolation Kit (cat. no., KIT0204, Invitrogen, USA), and cDNA was quantified by RT‒qPCR using a Roche Light Cycler 96 Real-time PCR system (Roche Diagnostics, Basel, Switzerland). The primers for real-time PCR were purchased from Generay Biotechnology Company, and the primer sequences are described in Table 2. The PCR conditions were as follows: 95 °C for 10 min, followed by 40 cycles of 95 °C for 15 s, 60 °C for 30 s and 72 °C for 30 s. The fold changes in the expression of genes (*Sirt1*, *SOD3, CAT, Nrf2, GPX4, Brca1, ATM* and *Msh2*) were calculated with the 2^−△△^CT method, with the housekeeping gene *β-actin* as the internal control.

### Statistical analysis

All data were analyzed by SPSS 20.0 software, and the metrological data were in accordance with a normal distribution and expressed as the mean ± SD. T-test was used to compare the two groups. One-way ANOVA was used to compare the mean of each group, different groups were compared using one-way analyses of variance followed by least significant difference (equal variances) or Games-Howell (unequal variances) post-hoc test. *p* < 0.05 was considered statistically significant.

**Table 2:** Primer sequences used for real-time PCR analysis

Gene (*Mus musculus*)	Forward primer (5’-3’)	Reverse primer (5’-3’)
*β-actin*	ACCTTCTACAATGAGCTGCG	CTGGATGGCTACGTACATGG
*Sirt1*	CTCTGAAAGTGAGACCAGTAGC	TGTAGATGAGGCAAAGGTTCC
*Brca1*	CGAATCTGAGTCCCCTAAAGAGC	AAGCAACTTGACCTTGGGGTA
*ATM*	GATCCTTCCCACTCCAGAAAC	ACTCCGCATAACTTCCATCG
*Msh2*	CCCAGGATGCCATTGTTAAAG	TACATAAGGAACGGGTGCTG
*SOD3*	CCTTCTTGTTCTACGGCTTGC	TCGCCTATCTTCTCAACCAGG
*CAT*	CCCCTATTGCCGTTCGATTCT	TTCAGGTGAGTCTGTGGGTTT
*Nrf2*	CTTTAGTCAGCGACAGAAGGAC	AGGCATCTTGTTTGGGAATGTG
*GPX4*	TGTGCATCCCGCGATGATT	CCCTGTACTTATCCAGGCAGA

## Supplementary Information

**Additional file 1.**
