# Supplementary material for: Effects of n-3 PUFA supplementation on oocyte in vitro maturation in mice with polycystic ovary syndrome
Source: J Ovarian Res. 2023 Apr 29;16:87. doi: 10.1186/s13048-023-01162-w (PMC10148539; doi:10.1186/s13048-023-01162-w)
Supplement: Supplementary file 1 — Additional file 1. [file 13048_2023_1162_MOESM1_ESM.docx]

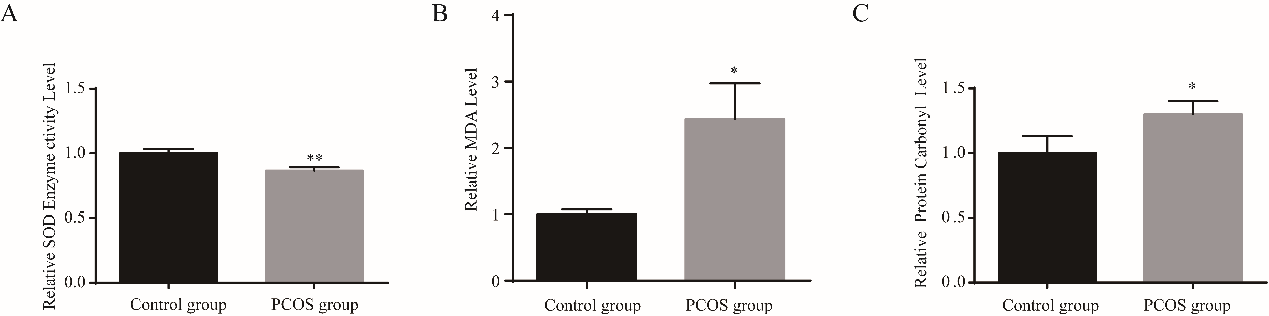


Supplementary Figure 1. The changes of oxidative stress markers in control and PCOS group ovaries. (A) the relative SOD Enzyme Activity level was measured by Total Superoxide Dismutase Assay Kit with WST-8 (cat. no., S0101, Beyotime, China); (B) the relative MDA level was measured by Lipid Peroxidation MDA Assay Kit (cat. no., S0131, Beyotime, China); (C) the relative Protein Carbonyl level was measured by Protein Carbonyl assay kit (cat. no., A087-1, Nanjing Jiancheng, China). Data are expressed as the mean ± SD. *, *p*<0.05. **, *p*<0.01.


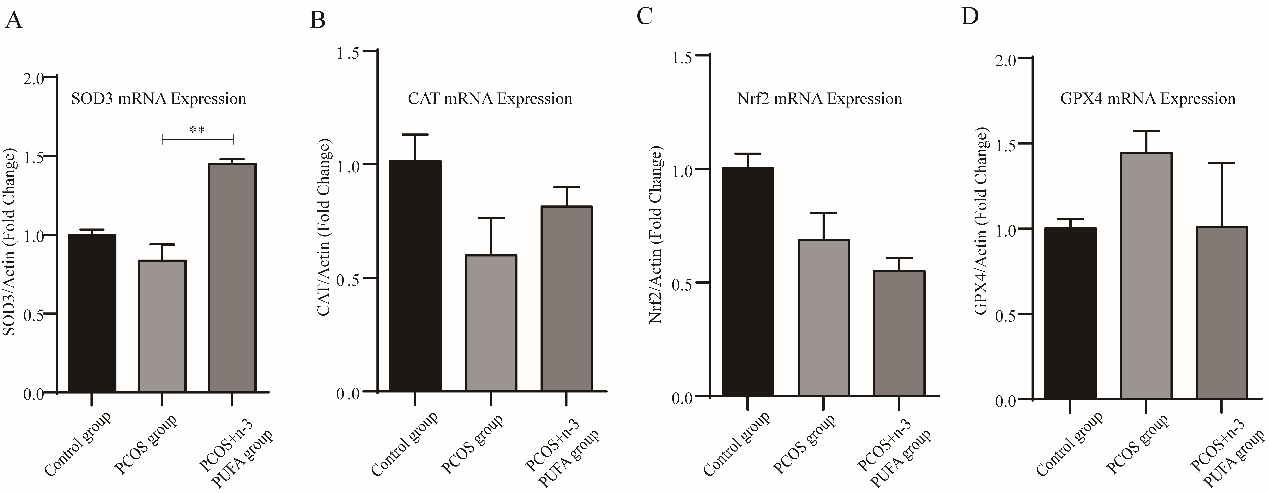


Supplementary Figure 2. The relative mRNA expression of *SOD3*, *CAT*, *Nrf2* and *GPX4* in control, PCOS and PCOS+n-3 PUFA group oocytes. The relative mRNA levels were determined by RT‐qPCR. Data are expressed as the mean ± SD. **, *p*<0.01.
